# Supplementary material for: Topology mediates transport of nanoparticles in macromolecular networks
Source: Nat Commun. 2022 Jul 14;13:4094. doi: 10.1038/s41467-022-31861-9 (PMC9283426; doi:10.1038/s41467-022-31861-9)
Supplement: Supplementary file 1 — Supplementry Information [file 41467_2022_31861_MOESM1_ESM.pdf]

Supplementary Information

Topology mediates transport of nanoparticles in macromolecular networks

Xiaobin Dai, Xuanyu Zhang, Lijuan Gao, ZiyangXu and Li-Tang Yan*

State Key Laboratory of Chemical Engineering, Department of Chemical Engineering, Tsinghua University, Beijing 100084, China

CONTENTS

I. Development of analytical model for free energy landscape [P2]

II. Minimal energy path of the transition of a particle between two neighboring cells [P6]

III. Development of analytical model for microscopic dynamics in different regimes [P9]

IV. Topological-mediated oscillation modes [P17]

V. Monte Carlo (MC) simulation [P19]

VI. Supporting figures [P22]

VII. Supplementary references [P32]

I. Development of analytical model for free energy landscape

To introduce the general formalism of the new analytical approach, we consider a hard particle of radius R in a cross-linked macromolecular network of Gaussian chains without dangling ends. The network topology is specified by (i) the set of cross-links $k=\{\mathbf{r}_i\}_{i=1}^{M}$,with M cross-links between the efficiently bridged Gaussian chains, (ii) the collection of linker connections marked as the tuple (i, j), and (iii) the continue curve path of linked strands $\mathbf{r}_{ij}(s)$ with contour variable $s\in[0,1]$. For a Gaussian chain of N bonds of Kuhn length b, the contour length $L = Nb$, and the average mesh size, $a_x = bN^{1/2} = 1$, is the unit length of the system. Considering the cross-linked chains in a dilute solution of θ-solvent, where screened excluded volume statistics can be assumed, the interaction potential between monomers is ignored to recover the ideal statistic[1]. Through coupling the excluded effect of the spherical nanoparticle into the renowned theory of network elasticity[2,3], the partition function of the particle-network system takes the form:

$$Z(\mathbf{r}_{np})=\prod_{k}\int d\mathbf{r}_k\prod_{(i,j)}\int D\mathbf{r}_{ij}\delta(\mathbf{r}_{ij}-\mathbf{r}_i)\delta(\mathbf{r}_{ij}-\mathbf{r}_j)\times\exp[-\beta\sum_{(i,j)}H(\mathbf{r}_{ij},\mathbf{r}_{np})] \tag{S1}$$

where $\mathbf{r}_k$ is the position vector of the cross-link point k, $\mathbf{r}_{ij} = \mathbf{r}_{ij}(s)$ is the path vector of the strand with its start $\mathbf{r}_{ij}(0) = \mathbf{r}_i$ and end $\mathbf{r}_{ij}(1) = \mathbf{r}_j$, $\mathbf{r}_{np}$ is the position vector of the nanoparticle, δ is the delta function, $\beta = 1/k_BT$, k_B is the Boltzmann constant, and T is the temperature. The Hamiltonian of the strand between cross-link pair (i, j) is given by,

$$H(\mathbf{r}_{ij},\mathbf{r}_{np})=\frac{3k_BT}{2Nb^2}\int_0^1 ds\left\|\frac{\partial\mathbf{r}_{ij}}{\partial s}\right\|^2+U_{pn}(\mathbf{r}_{ij},\mathbf{r}_{np}) \tag{S2}$$

Monomer interaction with the nanoparticle, $U_{pn}(\mathbf{r}_{ij}, \mathbf{r}_{np})$, is a hard-core potential,

$$U_{pn}(\mathbf{r}_{ij},\mathbf{r}_{np})=\begin{cases}\infty & \|\mathbf{r}_{ij}-\mathbf{r}_{np}\|<R\\ 0 & else\end{cases} \tag{S3}$$

In the absence of chain entanglement, all chains are set to be independently cross-linked, so that the partition function of the system is the product of all the partition functions of chains between cross-links (i, j), $Z(\mathbf{r}_{np})=\prod_{(i,j)} Z_{ij}(\mathbf{r}_i,\mathbf{r}_j,\mathbf{r}_{np})$, where the partition function of the cross-linked chain gives $Z_{ij}(\mathbf{r}_i,\mathbf{r}_j,\mathbf{r}_{np})=\int D\mathbf{r}_{ij}\delta(\mathbf{r}_{ij}(0)-\mathbf{r}_i)\delta(\mathbf{r}_{ij}(1)-\mathbf{r}_j)\times\exp[-\beta H(\mathbf{r}_{ij},\mathbf{r}_{np})]$, and $\int D\mathbf{r}_{ij}$ means the path integral over all conformations of the curve $\mathbf{r}_{ij}$. In the canonical ensemble, the Helmholtz free energy of the particle-network system is determined by

$$F(\mathbf{r}_{np})=-k_B T\ln Z(\mathbf{r}_{np})=\sum_{(i,j)} F_{ij}(\mathbf{r}_i,\mathbf{r}_j,\mathbf{r}_{np}) \tag{S4}$$

where $F_{ij}\left(\mathbf{r}_i,\mathbf{r}_j,\mathbf{r}_{np}\right)=-k_B T\ln Z_{ij}\left(\mathbf{r}_i,\mathbf{r}_j,\mathbf{r}_{np}\right)$.

As schemed in Fig. S9, we consider a spherical nanoparticle of radius R at the point O_0 ($\mathbf{r}_{np}=(x,y,z)$), and vectors $\mathbf{r}_i=(x_i,y_i,z_i)$ and $\mathbf{r}_j=(x_j,y_j,z_j)$ denote the position of cross-linked points i and j. The spatial position of segments on the curve at s is represented by the collection of three-dimensional vectors $\mathbf{r}$ = $\{\mathbf{r}_1,\mathbf{r}_2,\ldots,\mathbf{r}_N\}$, and the unit vector $\mathbf{u}_{ij}(s)$ = $d\mathbf{r}_{ij}(s)/ds$ determines the tangent direction. When the chain contacts with the spherical nanoparticle, the partition function of the strand can be rewritten as

$$Z_{ij}(\mathbf{r}_i,\mathbf{r}_j,\mathbf{r}_{np})=\int D\mathbf{r}_{ij}\delta(\mathbf{r}_{ij}(0)-\mathbf{r}_i)\delta(\mathbf{r}_{ij}(1)-\mathbf{r}_j)\exp\left\{-\frac{3}{2Nb^2}\int_0^1 ds\left\|\mathbf{u}_{ij}(s)\right\|^2\Phi(\left\|\mathbf{r}_{ij}-\mathbf{r}_{np}\right\|-R)\right\} \tag{S5}$$

where $\Phi(x)$ is the step unit function. For flexible chains, the correlation of the tangent vector $\mathbf{u}_{ij}(s)$ satisfies,

$$\int ds\mathbf{u}_{ij}(s)\bullet\mathbf{u}_{ij}(s')=\int ds\delta(s-s')\,||\,\mathbf{u}_{ij}(s)\,||^2 \tag{S6}$$

and the integral in Eq. S6 adopts the following form

$$\begin{aligned}&\int_0^1||\,\mathbf{u}_{ij}(s)\,||^2\,\Phi(||\,\mathbf{r}_{ij}-\mathbf{r}_{np}\,||-R)ds\\ =&\iint_{s,s'\in[0,1]^2}||\,\mathbf{u}_{ij}(s)\,||^2\,\Phi(||\,\mathbf{r}_{ij}-\mathbf{r}_{np}\,||-R)dsds'\\ =&\left(\int_0^1\mathbf{u}_{ij}(s)\Phi(||\,\mathbf{r}_{ij}-\mathbf{r}_{np}\,||-R)ds\right)^2\\ =&L_{ij}^2(\mathbf{r}_i,\mathbf{r}_j,\mathbf{r}_{np})\end{aligned} \tag{S7}$$

where $L_{ij}(\mathbf{r}_i,\mathbf{r}_j,\mathbf{r}_{np})=\int_0^1 \mathbf{u}_{ij}(s)\Phi(\|\mathbf{r}_{ij}-\mathbf{r}_{NP}\|-R)ds$ is the integral of the chain from a fixed end $\mathbf{r}_i$ to another one $\mathbf{r}_j$ as to the position of the nanoparticle $\mathbf{r}_{np}$,

To calculate the integral of a strand $L_{ij}(\mathbf{r}_i,\mathbf{r}_j,\mathbf{r}_{np})$ cross-linked by points P and Q with positional vectors $\mathbf{r}_\mathrm{i}$ and $\mathbf{r}_\mathrm{j}$, we consider an osculating plane A coming across the points P and Q with the normal vector $\mathbf{n}$. As shown in Fig. S10, the path of the chain $\mathbf{r}_{ij}(s)$ from P to Q in the plane A desires to be one of the conformations of chains. Randomly selecting the normal vector $\mathbf{n}$ gives all conformations of chains that slip around the spherical surface of nanoparticle. For the sake of discussing the geometric relations of the sphere and the plane A for any vector $\mathbf{n}$, it can be found: (i) the point U which is perpendicular to PQ, measuring the particle-strand distance l

$$l=O_0U=\frac{\left|(\mathbf{r}_i-\mathbf{r}_{np})\times(\mathbf{r}_j-\mathbf{r}_{np})\right|}{\left|\mathbf{r}_i-\mathbf{r}_j\right|} \tag{S8}$$

where $l\le\min\left\{\left\|\mathbf{r}_i-\mathbf{r}_{np}\right\|,\left\|\mathbf{r}_j-\mathbf{r}_{np}\right\|\right\}=R_f$; (ii) the projection point O_1 of the point O_0 in the plane A, determining the distance from the point O_0 to the plane A:

$$R_s=O_0O_1=l\cos\alpha \tag{S9}$$

where the dihedral angle $\angle O_0UO_1=\alpha\in[0,\pi)$, and $R_s\le l\le R_f$. In particular, three cases of the problem are studied:

(i) If $R\le R_s$, the sphere and plane have one tangent point at most, so that the integral is equal to the mesh size, giving

$$L_{ij}(\mathbf{r}_i,\mathbf{r}_j,\mathbf{r}_{np})=a_x=bN^{1/2} \tag{S10}$$

(ii) If $R_s<R<R_f$, the sphere and the plane A have intersecting circle C, and the radius of intersected circle is:

$$R_c=\sqrt{R^2-l^2} \tag{S11}$$

In this case, we divide the curve into two line segments PT_1, QT_2 and two arcs T_1T_0, T_0T_2 by tangent points T_0, T_1, and T_2. We then obtain the integral from point P to point Q by geometrical relationship,

$$L_{ij}(\mathbf{r}_i,\mathbf{r}_j,\mathbf{r}_{np}) = \overline{PT_1} + T_1T_0 + T_0T_2 + \overline{QT_2} \tag{S12}$$

According to the geometric relationship, the lengths are given in the following,

$$\overline{PT_1} = \sqrt{(\mathbf{r}_i - \mathbf{r}_{np})^2 - R^2}$$

$$\overline{QT_2} = \sqrt{(\mathbf{r}_j - \mathbf{r}_{np})^2 - R^2}$$

$$T_1T_0 = R_c(\arccos\sqrt{\frac{l^2 - l^2\sin\alpha}{(\mathbf{r}_i - \mathbf{r}_{np})^2 - l^2\sin\alpha}} - \arccos\sqrt{\frac{R_c^2}{(\mathbf{r}_i - \mathbf{r}_{np})^2 - l^2\sin\alpha}})$$

$$T_0T_2 = R_c(\arccos\sqrt{\frac{l^2 - l^2\sin\alpha}{(\mathbf{r}_j - \mathbf{r}_{np})^2 - l^2\sin\alpha}} - \arccos\sqrt{\frac{R_c^2}{(\mathbf{r}_j - \mathbf{r}_{np})^2 - l^2\sin\alpha}})$$

Hence, the solution of Eq. S12 can be written as

$$L_{ij}(\mathbf{r}_i,\mathbf{r}_j,\mathbf{r}_{NP}) = \sum_m^{i,j}\left\{\sqrt{(\mathbf{r}_m - \mathbf{r}_{np})^2 - R^2} + R_c\left[\arccos\sqrt{\frac{l^2 - l^2\sin^2\alpha}{(\mathbf{r}_m - \mathbf{r}_{np})^2 - l^2\sin^2\alpha}} - \arccos\sqrt{\frac{R_c^2}{(\mathbf{r}_m - \mathbf{r}_{np})^2 - l^2\sin^2\alpha}}\right]\right\} \tag{S13}$$

(iii) If $R \geq R_f$, cross-linked points will be reflected from boundary surface using bounce-back rule[4] once they are found inside the spherical surface. New positions on the nanoparticle surface are taken to be projection of the nanoparticle position on its surface, which takes the following transformation,

$$\mathbf{r}_m \leftarrow R\frac{\mathbf{r}_m - \mathbf{r}_{np}}{\left|\mathbf{r}_m - \mathbf{r}_{np}\right|} + \mathbf{r}_{np} \qquad (r_m \leq R) \tag{S14}$$

where $m = i$ or j. Therefore, Eq. S13 can be transformed into

$$L_{ij}(\mathbf{r}_i,\mathbf{r}_j,\mathbf{r}_{np}) = \sum_m^{i,j} R_c \arccos\sqrt{\frac{l^2 - l^2\sin^2\alpha}{R_c^2}} \tag{S15}$$

II. Minimal energy path of the transition of a nanoparticle between two neighboring cells

In this section, we provide a detailed evidence regarding that the linear connection between the core region of a network cell and the local free energy minimum at the center of a cell face, which is defined as z axis in the main text, is one of the minimum energy paths (MEPs).

As schemed in Fig. S9, we still consider a spherical nanoparticle of radius R at the point O_0 ($\mathbf{r}_{np}=(x,y,z)$), and vectors $\mathbf{r}_i=(x_i,y_i,z_i)$ and $\mathbf{r}_j=(x_j,y_j,z_j)$ denote the position of cross-linked points i and j. For a Platonic or Archimedean polyhedral[5], z axis has one rotational symmetry axis C_n, and the rotation matrix of symmetry operation $\mathbf{C}_n^k$ is

$$\mathbf{C}_n^k=\begin{pmatrix} \cos\frac{2\pi}{n} & \sin\frac{2\pi}{n} & 0 \\ -\sin\frac{2\pi}{n} & \cos\frac{2\pi}{n} & 0 \\ 0 & 0 & 1 \end{pmatrix}^k \tag{S16}$$

where the rotation matrix $\mathbf{C}_n^k$ denotes rotations of $2\pi/n$, performed k times. Using the rotation matrix, we can extract a group of chains, p, with the positions of the chain ends $\{\mathbf{r}_{ik}\}$, $\{\mathbf{r}_{jk}\}$ given by $\mathbf{r}_{i_k}=C_n^k\mathbf{r}_{i_1}, \mathbf{r}_{j_k}=C_n^k\mathbf{r}_{j_1}$. Using the Eq. S4, the free energy of the total chains in group p is given by

$$F=\sum_{i_k,j_k\in p} F_{i_kj_k}(\mathbf{r}_{i_k},\mathbf{r}_{j_k},\mathbf{r}_{np})=\sum_{k=1}^{n}F_{i_1j_1}(\mathbf{r}_{i_1},\mathbf{r}_{j_1},\mathbf{C}_n^{-k}\mathbf{r}_{np}) \tag{S17}$$

Now we prove that for any point along z axis, $\mathbf{r}_{np}=(0,0,z)$, the profiles of free energy $F(\mathbf{r}_{np})$ satisfy the saddle point condition, i.e., the differential of free energy $\Delta F(\mathbf{r}_{np})=F(\mathbf{r}_{np}+\Delta\mathbf{r}_{np})-F(\mathbf{r}_{np})=0$, where $\Delta\mathbf{r}_{np}=(\delta x,\delta y,0)$ is a small displacement perpendicular to z axis. For this purpose, we consider that the small displacement $\Delta\mathbf{r}_{np}$ is given

to a nanoparticle at a position along z axis, the new position can be written as $\mathbf{r}'_{np} = \mathbf{r}_{np} + \Delta\mathbf{r}_{np}$.

Using Eq. S16, we get

$$C_n^{-k}\mathbf{r}'_{np} = C_n^{-k}(\mathbf{r}_{np} + \Delta\mathbf{r}_{np}) = \mathbf{r}_{np} + C_n^{-k}\Delta\mathbf{r}_{np}$$

$$\sum_{k=1}^{n} C_n^{-k}\Delta\mathbf{r}_{np} = \mathbf{0} \tag{S18}$$

By substituting Eq. S18 into Eq. S17, the differential of the free energy of chains can be calculated as

$$\begin{aligned}\Delta F &= \sum_{k=1}^{n}\left[F_{i_1 j_1}(\mathbf{r}_{i_1}, \mathbf{r}_{j_1}, \mathbf{C}_n^{-k}(\mathbf{r}_{np} + \Delta\mathbf{r}_{np})) - F_{i_1 j_1}(\mathbf{r}_{i_1}, \mathbf{r}_{j_1}, \mathbf{C}_n^{-k}\mathbf{r}_{np})\right] \\ &\approx \frac{3k_B T}{Nb^2} L_{ij}(\mathbf{r}_i, \mathbf{r}_j, \mathbf{r}_{np}) \sum_{k=1}^{n} \Delta L_{ij}(\mathbf{r}_i, \mathbf{r}_j, \mathbf{C}_n^{-k}\mathbf{r}_{np})\end{aligned} \tag{S19}$$

where $L_{ij}(\mathbf{r}_i, \mathbf{r}_j, \mathbf{r}_{np})$ represents the minimal value of the path integral of the curve $\mathbf{r}_{ij}$, and $\Delta L_{ij}(\mathbf{r}_i, \mathbf{r}_j, \mathbf{r}_{np}) = L_{ij}(\mathbf{r}_i, \mathbf{r}_j, \mathbf{r}_{np} + \Delta\mathbf{r}_{np}) - L_{ij}(\mathbf{r}_i, \mathbf{r}_j, \mathbf{r}_{np})$ is its differential.

In combination with Eqs. S10, S13 and S15, the minimal value of the integral $L_{ij}(\mathbf{r}_i, \mathbf{r}_j, \mathbf{r}_{np})$ is obtained by setting the dihedral angle α=0, taking the form

$$L_{ij}(\mathbf{r}_i, \mathbf{r}_j, \mathbf{r}_{np}) = \begin{cases} bN^{1/2} & R \le l \\ \sum_{m}^{i,j}\left\{\sqrt{(\mathbf{r}_m - \mathbf{r}_{np})^2 - R^2} + R\left[\arccos\frac{l}{|\mathbf{r}_m - \mathbf{r}_{np}|} - \arccos\frac{R}{|\mathbf{r}_m - \mathbf{r}_{np}|}\right]\right\} & l < R < R_f \\ \sum_{m}^{i,j} R\arccos\frac{l}{R} & R \ge R_f \end{cases} \tag{S20}$$

where $R_f = \min\left\{\left\|\mathbf{r}_i - \mathbf{r}_{np}\right\|, \left\|\mathbf{r}_j - \mathbf{r}_{np}\right\|\right\}$. Now we turn to discuss the values of sum of the differentials $\sum_{k=1}^{n} \Delta L_{ij}(\mathbf{r}_i, \mathbf{r}_j, \mathbf{C}_n^{-k}\mathbf{r}_{np})$ in the different regimes denoted in Eq. S20.

(1) For $R \le l$, the differential of Eq. S20 is given by

$$\Delta L(\mathbf{r}_i,\mathbf{r}_j,\mathbf{r}_{np}) = L(\mathbf{r}_i,\mathbf{r}_j,\mathbf{r}_{NP}+\Delta\mathbf{r}_{np}) - L(\mathbf{r}_i,\mathbf{r}_j,\mathbf{r}_{np}) = 0 \qquad \text{(S21)}$$

and $\sum_{k=1}^{n}\Delta L(\mathbf{r}_i,\mathbf{r}_j,\mathbf{C}_n^{-k}\mathbf{r}_{np}) = 0$.

(2) For $l < R < R_f$, the differential of Eq. S20 takes the form:

$$\Delta L(\mathbf{r}_i,\mathbf{r}_j,\mathbf{r}_{np}) = \sum_{m}^{i,j}(\mathbf{M}_1+\mathbf{M}_2+\mathbf{M}_3)\Delta\mathbf{r}_{np} \qquad \text{(S22)}$$

where the vectors $\mathbf{M}_1$, $\mathbf{M}_2$, $\mathbf{M}_3$ are

$$\mathbf{M}_1 = \left(\frac{1}{\sqrt{(\mathbf{r}_m-\mathbf{r}_{np})^2-R^2}} + \frac{R^2}{\left|\mathbf{r}_m-\mathbf{r}_{np}\right|^2\sqrt{(\mathbf{r}_m-\mathbf{r}_{np})^2-R^2}}\right)(\mathbf{r}_{np}-\mathbf{r}_m)$$

$$\mathbf{M}_2 = \frac{l}{\left|\mathbf{r}_m-\mathbf{r}_{np}\right|\sqrt{\left|\mathbf{r}_m-\mathbf{r}_{np}\right|^2-l^2}}(\mathbf{r}_{np}-\mathbf{r}_m) \qquad \text{(S23)}$$

$$\mathbf{M}_3 = \frac{R}{\sqrt{\left|\mathbf{r}_m-\mathbf{r}_{np}\right|^2-l^2}}\frac{\left[(\mathbf{r}_i-\mathbf{r}_{np})\times(\mathbf{r}_j-\mathbf{r}_{np})\right]\times(2\mathbf{r}_{np}-\mathbf{r}_i-\mathbf{r}_j)}{\left|\mathbf{r}_i-\mathbf{r}_j\right|\left|(\mathbf{r}_i-\mathbf{r}_{np})\times(\mathbf{r}_j-\mathbf{r}_{np})\right|}$$

By substituting Eq. S18 to Eq. S22, the differential of free energy is given by

$$\sum_{k=1}^{n}\Delta L(\mathbf{r}_i,\mathbf{r}_j,\mathbf{C}_n^{-k}\mathbf{r}_{np}) = \sum_{m}^{i,j}\left\{\mathbf{M}_l+\mathbf{M}_2+\mathbf{M}_3\right\}\sum_{k=1}^{n}\mathbf{C}_n^{-k}\Delta\mathbf{r}_{np} = 0 \qquad \text{(S24)}$$

(3) For $R \geq R_f$, the differential of Eq. S20 can be written as:

$$\Delta L(\mathbf{r}_i,\mathbf{r}_j,\mathbf{r}_{np}) = \sum_{m}^{i,j} R\frac{\left[(\mathbf{r}_i-\mathbf{r}_{np})\times(\mathbf{r}_j-\mathbf{r}_{np})\right]\times(\mathbf{r}_i+\mathbf{r}_j-2\mathbf{r}_{np})}{\left|\mathbf{r}_i-\mathbf{r}_j\right|\left|(\mathbf{r}_i-\mathbf{r}_{np})\times(\mathbf{r}_j-\mathbf{r}_{np})\right|\sqrt{1-(l/R)^2}}\cdot\Delta\mathbf{r}_{np} \qquad \text{(S25)}$$

Substituting Eq. S18 into Eq. S25**,** the differential is given by

$$\sum_{i=1}^{n}\Delta L(\mathbf{r}_i,\mathbf{r}_j,\mathbf{C}_n^{-k}\mathbf{r}_{np}) = \sum_{m}^{i,j} R\frac{\left[(\mathbf{r}_i-\mathbf{r}_{np})\times(\mathbf{r}_j-\mathbf{r}_{np})\right]\times(\mathbf{r}_i+\mathbf{r}_j-2\mathbf{r}_{np})}{\left|\mathbf{r}_i-\mathbf{r}_j\right|\left|(\mathbf{r}_i-\mathbf{r}_{np})\times(\mathbf{r}_j-\mathbf{r}_{np})\right|\sqrt{1-(l/R)^2}}\cdot\sum_{k=1}^{n}\mathbf{C}_n^{-k}\Delta\mathbf{r}_{np} = 0 \qquad \text{(S26)}$$

Clearly, $\sum_{k=1}^{n} \Delta L(\mathbf{r}_i, \mathbf{r}_j, \mathbf{C}_n^{-k} \mathbf{r}_{np}) = 0$ keeps in all regimes. Hence, the differential of free energy can be expressed as

$$\Delta F \approx \frac{3k_B T}{Nb^2} L(\mathbf{r}_i, \mathbf{r}_j, \mathbf{r}_{np}, 0) \sum_{k=1}^{n} \Delta L(\mathbf{r}_i, \mathbf{r}_j, \mathbf{C}_n^{-k} \mathbf{r}_{np}, 0) = 0 \qquad \text{(S27)}$$

This clarifies that any point on z-axis is saddle point, corroborating that z-axis is one of the MEPs.

III. Development of analytical model for microscopic dynamics in different regimes

The microscopic dynamics of the nanoparticle is theoretically well described by a nonlinear Langevin equation[6],

$$m \frac{d^2 z}{dt^2} = -\Delta F'(z) - \gamma \frac{dz}{dt} + \xi(t) \qquad \text{(S28)}$$

where $\Delta F(z)$ is the potential of the nanoparticle at position z, m is the mass of nanoparticle, γ is the friction coefficient, and $\xi(t)$ is the fluctuating force. According to the fluctuation-dissipative theorem, the fluctuating force satisfies $\langle \xi(t) = 0 \rangle$, $\langle \xi(t)\xi(t') = 2\gamma k_B T \delta(t - t') \rangle$, and $\langle ... \rangle$ denotes the ensemble average over many trajectories. Here, we analyze the one-dimensional diffusion along its reaction coordinate z through examining the mean square displacement (MSD) $\langle \Delta z^2(t) \rangle$ for the nanoparticle in different regimes.

In order to extract the dynamics described by Eq. S28 in the periodic potentials of Regimes I, II and III, we need to coarse-grain and decompose it into a series of consecutive jump and waiting events, where the nanoparticle spends most of the time close to a cell center and only occasionally escapes to another one. We identify these trapping periods with the waiting times

and the escape events with the jumps of the continuous time random walk (CTRW)[7]. It has to wait for a random waiting time drawn from the probability distribution function (PDF) of the waiting time $\psi(t)$, before it makes a jump, and the length of the jump can be chosen to be a random variable, δz, distributed in terms of the PDF, $\lambda(\delta z)$. For the present discussion, we assume that the CTRW process is separable, in the sense that t and δz are independently identically distribution (IID). The position of the nanoparticle is given by,

$$z(t) = \sum_{i=0}^{N(t)} \delta z_i(t) \tag{S29}$$

where $N(t)$ is the counts of transition events at time t, and δz_i is the jump length of the event i. We define $P(N, t)$ as the probability of N events occurring in time t, and

$$P(N,t) = \psi(t)^N P(0,t) \tag{S30}$$

Taking the Laplace transform of Eq. S30,

$$P(N,s) = \psi(s)^N \frac{1-\psi(s)}{s} \tag{S31}$$

where $\psi(s)$ represents the Laplace transformation of $\psi(t)$, and s is a complex variable in Laplace domain. Based on Eq. S31, we can derive the first and second moments of $N(t)$ in Laplace domain, which take the forms:

$$\langle N(s) \rangle = \sum_{N=0}^{\infty} NP(N,s) = \frac{\psi(s)}{s(1-\psi(s))} \tag{S32}$$

$$\langle N^2(s) \rangle = \sum_{N=0}^{\infty} N^2 P(N,s) = 2\frac{\psi(s)^2}{s(1-\psi(s))^2} + \frac{\psi(s)}{s(1-\psi(s))} \tag{S33}$$

The distribution of the total number of network cells traversed can accordingly be obtained from the Montroll-Weiss equation[7],

$$P(k,s)=\sum_{N=0}^{\infty}P(N,s)P_N(k)=\frac{1-\psi(s)}{s}\frac{1}{1-\psi(s)\lambda(k)} \tag{S34}$$

where $P(k, s)$ is the Fourier-Laplace transformation of displacement PDF $P(z, t)$, $\lambda(k)$ is the Fourier transformation of $\lambda(\delta z)$, and k is a complex variable in Fourier space. Using Eq. S34, we can calculate the expected position of the nanoparticle $z(t)$ in the Laplace domain

$$\langle z(s)\rangle=\frac{1}{i}\frac{dP(k,s)}{ds}\bigg|_{k=0}=\frac{\psi(s)}{s(1-\psi(s))}\langle\delta z(s)\rangle \tag{S35}$$

$$\langle z^2(s)\rangle=\frac{1}{i^2}\frac{d^2P(k,s)}{ds^2}\bigg|_{k=0}=\frac{\psi(s)}{s(1-\psi(s))}\langle\delta z^2(s)\rangle+2\frac{\psi(s)}{s(1-\psi(s))^2}\langle\delta z(s)\rangle^2 \tag{S36}$$

Substituting Eqs. S32 and S33 into Eq. S36 and performing the inverse Laplace transformation, we can get the second moment of the total displacement $\Delta z(t) = z(t) - z(0)$,

$$\langle\Delta z^2(t)\rangle=\langle N(t)\rangle\left(\langle\delta z^2(t)\rangle-\langle\delta z(t)\rangle^2\right)+\langle N^2(t)\rangle\langle\delta z(t)\rangle^2 \tag{S37}$$

where $\delta z{=}\delta z(t)\in[-r_{\text{in}},r_{\text{in}})$ is the displacement of nanoparticle in a network cell, r_{in} is the radius of the inscribed sphere of the cell, and $\langle\delta z(t)\rangle=0$ gives the α-stable distribution of the displacement.

In order to accurately predict the trajectory of the nanoparticle, a waiting time distribution $\psi(t)$ can be obtained in exponential distribution via Poisson processes

$$\psi(t)=\frac{1}{t_w}\exp(-\frac{t}{t_w}) \tag{S38}$$

Combining Eqs. S32 and S38 and performing inverse Laplace transformation, the average number of jumps gives $\langle N(t)\rangle=t/t_w$. Therefore, Eq. S37 can be simplified as

$$\left\langle \Delta z^2(t) \right\rangle = \begin{cases} \frac{t}{t_w} \left\langle \delta z^2(t) \right\rangle & t \geq t_w \\ \left\langle \delta z^2(t) \right\rangle & t < t_w \end{cases} \tag{S39}$$

The characteristic waiting time t_w can be determined by Kramers' rate theory[8],

$$t_w = \frac{2\pi\gamma}{\sqrt{|k_s|k_l}} \exp(U_b / k_B T) \tag{S40}$$

where k_s and k_l are the curvatures of the potential along z-axis at the minimum point $z = 0$ and the maximum point $z = r_{\mathrm{in}}$, respectively.

In view of Eq. S39, for the long time scale, MSD recovers back to the normal diffusion. For the short time scale with $t < t_w$, $\left\langle \Delta z^2(t) \right\rangle = \left\langle \delta z^2(t) \right\rangle$, indicating that the nanoparticle displacement is equivalent to the displacement of a nanoparticle in a single cell, where hopping events don't happen and it is unnecessary to distinguish between δz and z. In the latter case, the microscopic dynamics of the nanoparticle in different regimes will significantly depend on the form of the energy landscape, which is discussed as follow:

Regime I: The potential $\Delta F(z) = \rho|z|$ and is "V-shaped", with $\rho=U_b/r_{\mathrm{in}}$ being an arbitrary constant[9]. Thus, for a nanoparticle diffusing in a potential $F(z)$ the Fokker–Planck equation describing the dynamics of the PDF $P(z, t)$ under a constant resetting rate $r=1/t_w$ reads

$$\frac{\partial}{\partial t} P(z,t) = \left(\frac{\partial}{\partial z} F'(z) + D_0 \frac{\partial^2}{\partial z^2} \right) P(z,t) - rP(z,t) + r\delta(z - z_0) \tag{S41}$$

where $D_0 = k_B T/6\pi R\gamma$ represents the diffusion coefficient of the nanoparticle in solvents. Taking the Laplace transformation of Eq. S41,

$$sP(z,s) - P(z,0) = \left(\frac{\partial}{\partial z} F'(z) + D_0 \frac{\partial^2}{\partial z^2} \right) P(z,s) - rP(z,s) \tag{S42}$$

The solution to the above equation reads

$$P(z,s)=\begin{cases} a_+ e^{m_+ z} & z<0 \\ b_+ e^{-m_+ z}+b_- e^{-m_- z} & 0\le z\le z_0 \\ c_+ e^{-m_+ z} & z>z_0 \end{cases} \tag{S43}$$

where $m_\pm=\rho(1\pm\Delta_{r+s})/2D_0$ are the roots of the auxiliary equation $m^2-(\rho/D_0)m-(r+s)/D_0=0$, and $\Delta_{r+s}=\sqrt{1+4D_0(r+s)/\rho^2}$. Now, continuity of the solution at the points z = 0 and z = z_0 fixes respectively two relations between the parameters, $a_+=b_++b_-$ and $c_+=b_++b_-\exp\left(|m_+-m_-|z_0\right)$.

In addition to the continuity of the solution at the resetting point and turning point of the free energy, the derivative of $P(z, s)$ exhibits a discontinuity at the two points. Firstly, the discontinuity at the origin arises due to the singular nature of the potential at the turning point and is obtained by integrating Eq. S42, yielding

$$\left(\frac{\partial}{\partial z}P(z,s)\bigg|_{z=0^+}-\frac{\partial}{\partial z}P(z,s)\bigg|_{z=0^-}\right)+\frac{1}{D_0}\left(F'(z)P(z,s)\big|_{z=0^+}-F'(z)P(z,s)\big|_{z=0^-}\right)=0 \tag{S44}$$

Such that,

$$2\left(m_+-\frac{\rho}{D_0}\right)b_+=\left(\frac{2\rho}{D_0}-m_+-m_-\right)b_- \tag{S45}$$

At t=0^+, the starting condition of Eq. S42 can be written as

$$\left(\frac{\partial}{\partial z}P(z,s)\bigg|_{z=z_0^+}-\frac{\partial}{\partial z}P(z,s)\bigg|_{z=z_0^-}\right)+\frac{1}{D_0}\left(F'(z)P(z,s)\big|_{z=z_0^+}-F'(z)P(z,s)\big|_{z=z_0^-}+\frac{r+s}{sD_0}\right)=0 \tag{S46}$$

Thus, the solution of the parameter b_- reads

$$b_-=\frac{r+s}{sD_0}\frac{e^{m_- x_0}}{m_+-m_-} \tag{S47}$$

Inserting values of the parameters in Eq. S43, we have the solution for the PDF under resetting in Laplace domain

$$P(z,s)=\begin{cases}\dfrac{r+s}{s}\dfrac{1}{\rho\left(\Delta_{r+s}-1\right)}\exp\left(\dfrac{\rho}{2D_0}\left[(z+z_0)+\Delta_{r+s}(z-z_0)\right]\right) & z\le 0\\ \dfrac{r+s}{s}\dfrac{1}{\rho\Delta_{r+s}\left(\Delta_{r+s}-1\right)}\exp\left(\dfrac{\rho}{2D}\left[z_0-\Delta_{r+s}z_0-z\right]\right)\left[\Delta_{r+s}\exp\left(\dfrac{\rho}{2D_0}z\Delta_{r+s}\right)-2\sinh\left(\dfrac{\rho}{2D_0}z\Delta_{r+s}\right)\right] & 0<z<z_0\\ \dfrac{r+s}{s}\dfrac{1}{\rho\Delta_{r+s}\left(\Delta_{r+s}-1\right)}\exp\left(\dfrac{\rho}{2D}\left[z_0-\Delta_{r+s}z-z\right]\right)\left[\Delta_{r+s}\exp\left(\dfrac{\rho}{2D_0}z_0\Delta_{r+s}\right)-2\sinh\left(\dfrac{\rho}{2D_0}z_0\Delta_{r+s}\right)\right] & z\ge z_0\end{cases} \tag{S48}$$

From the solution Eq. S48, we can calculate the MSD $\left\langle z^2(t)\right\rangle=\int_{-\infty}^{+\infty}z^2P(z,t)dz$, which in the Laplace domain reads

$$\begin{aligned}\left\langle z^2(s)\right\rangle&=\int_{-\infty}^{0}z^2P(z,s)dz+\int_{0}^{z_0}z^2P(z,s)dz+\int_{z_0}^{+\infty}z^2P(z,s)dz\\&=\frac{z_0^2}{s}+\frac{2(D_0-\rho z_0)}{s(r+s)}-\frac{\rho^2}{s(r+s)^2}\exp\left(\frac{\rho z_0(1-\Delta_{r+s})}{2D_0}\right)\left[1+\Delta_{r+s}-2\exp\left(-\frac{\rho z_0(1-\Delta_{r+s})}{2D_0}\right)\right]\end{aligned} \tag{S49}$$

For the symmetric case (z_0 = 0), Eq. S49 reduces to the expression

$$\left\langle z^2(s)\right\rangle=\frac{8D_0^2}{\rho^2}\frac{1}{s\left(1+\sqrt{1+\dfrac{4D_0(r+s)}{\rho^2}}\right)^2} \tag{S50}$$

Given that the characteristic equation $s(1+\sqrt{1+4D_0(r+s)/\rho^2})^2=0$ has a complex root $s_1=(-\rho^2/4D_0-r)+(\rho^2/4D_0)i$, the Laplace transformation of Eq. S50 has the form $\left\langle\Delta z^2(t)\right\rangle\sim e^{s_1t}$ at intermediate time scale ($t\to 1/|s_1|$), following an underdamped mode of oscillation (See section IV for more information). At long time scale ($t\to\infty$), the MSD in Regime I saturates to a constant, given by

$$\left\langle\Delta z^2(t)\right\rangle\sim 2D_0t_w-\rho^2t_w^2(\Delta_0-1) \tag{S51}$$

where $\Delta_0=\sqrt{1+4D_0/\left(\rho^2t_w\right)}$.

Regime II: With $\Delta F(z) = \omega^2 z^2 / 2$, we proceed to apply Langevin's original strategy to study the problem of the harmonic oscillator motion, where $\omega = (2\Delta U_b)^{1/2} / r_{\text{in}}$ is the frequency of the harmonic oscillator[10]. Here, we calculate the MSD for the nanoparticle bounded by the harmonic potential, for which the Langevin Eq. S28 can be written as

$$\frac{d^2 z}{dt^2} + \frac{\gamma}{m}\frac{dz}{dt} + \omega^2 z = \frac{1}{m}\xi(t) \tag{S52}$$

Taking the ensemble average[11] with the hypothesis $\langle \xi(t) z(t) \rangle = 0$, the Eq. S52 can be transformed into

$$\frac{d^2\langle z^2(t)\rangle}{dt^2} - 2\langle v(t)\rangle^2 + 2\frac{\gamma}{m}\frac{d\langle z^2(t)\rangle}{dt} + 2\omega^2\langle z^2(t)\rangle = 0 \tag{S53}$$

where $v(t) = dz/dt$ represents the velocity of the nanoparticle. We apply the energy equipartition theorem, $\langle v(t)\rangle = 0$, $m\langle v^2(t)\rangle / 2 = k_B T / 2$, to the Eq. S53, implying that

$$\frac{d^2\langle z^2(t)\rangle}{dt^2} + \frac{\gamma}{m}\frac{d\langle z^2(t)\rangle}{dt} + 2\omega^2\langle z^2(t)\rangle = 2\frac{k_B T}{m} \tag{S54}$$

To solve the second-order differential equation Eq. S54, we firstly solve the homogeneous equation given by

$$\frac{d^2\langle z^2(t)\rangle}{dt^2} + \frac{\gamma}{m}\frac{d\langle z^2(t)\rangle}{dt} + 2\omega^2\langle z^2(t)\rangle = 0 \tag{S55}$$

which can be classified into two conditions:

(a) In the overdamped case ($\gamma > \sqrt{8} m\omega$), the homogeneous solution reads

$$\langle z^2(t)\rangle = C_1 e^{(-\frac{\gamma}{m} - \frac{1}{2}\sqrt{(\frac{\gamma}{m})^2 - 8\omega^2})t} + C_2 e^{(-\frac{\gamma}{m} + \frac{1}{2}\sqrt{(\frac{\gamma}{m})^2 - 8\omega^2})t} \tag{S56}$$

With a constant particular solution $\langle z^2(t)\rangle = k_B T / m\omega^2$, the complete solution of Eq. S54 can be written as

$$\left\langle z^2(t)\right\rangle = C_1 e^{(-\frac{\gamma}{m}-\frac{1}{2}\sqrt{(\frac{\gamma}{m})^2-8\omega^2})t} + C_2 e^{(-\frac{\gamma}{m}+\frac{1}{2}\sqrt{(\frac{\gamma}{m})^2-8\omega^2})t} + \frac{k_B T}{m\omega^2} \quad \text{(S57)}$$

Through the initial conditions $\left\langle z^2(t)\right\rangle = 0$, $\frac{d\left\langle z^2(t)\right\rangle}{dt} = 0$ at t=0, the exact values of constants C_1 and C_2 in Eq. S57 are determined as

$$\begin{aligned} C_1 &= \frac{k_B T\gamma}{2m^2\omega^2\sqrt{(\frac{\gamma}{m})^2 - 8\omega^2}} - \frac{k_B T}{2m\omega^2} \\ C_2 &= -\frac{k_B T\gamma}{2m^2\omega^2\sqrt{(\frac{\gamma}{m})^2 - 8\omega^2}} - \frac{k_B T}{2m\omega^2} \end{aligned} \quad \text{(S58)}$$

Such that the Eq. S57 can be transformed into

$$\left\langle z^2(t)\right\rangle = \frac{k_B T}{m\omega^2}\left[1 - e^{-\frac{\gamma t}{2m}}\left(2\sinh^2(\frac{1}{4}\beta_1 t) + \frac{\gamma}{m\beta_1}\sinh(\frac{1}{2}\beta_1 t) + 1\right)\right] \quad \text{(S59)}$$

where $\beta_1 = \sqrt{(\frac{\gamma}{m})^2 - 8\omega^2}$.

(b) In the underdamped case ($\gamma < \sqrt{8}m\omega$), the solution reads

$$\left\langle z^2(t)\right\rangle = \frac{k_B T}{m\omega^2}\left[1 - e^{-\frac{\gamma t}{2m}}\left(-2\sin^2(\frac{\sqrt{2}}{2}\omega_1 t) + \frac{\gamma}{\sqrt{8}m\omega_1}\sin(\sqrt{2}\omega_1 t) + 1\right)\right] \quad \text{(S60)}$$

where $\omega_1 = \sqrt{\omega^2 - \frac{1}{8}(\frac{\gamma}{m})^2}$. We set the initial condition $z(0) = 0$, and thus the MSD in Regime II gives

$$\left\langle \Delta z(t)^2\right\rangle = \begin{cases} \frac{k_B T}{m\omega^2}\left[1 - e^{-\frac{\gamma t}{2m}}\left(2\sinh^2(\frac{1}{4}\beta_1 t) + \frac{\gamma}{m\beta_1}\sinh(\frac{1}{2}\beta_1 t) + 1\right)\right] & \gamma > \sqrt{8}m\omega \\ \frac{k_B T}{m\omega^2}\left[1 - e^{-\frac{\gamma t}{2m}}\left(-2\sin^2(\frac{\sqrt{2}}{2}\omega_1 t) + \frac{\gamma}{\sqrt{8}m\omega_1}\sin(\sqrt{2}\omega_1 t) + 1\right)\right] & \gamma < \sqrt{8}m\omega \end{cases} \quad \text{(S61)}$$

Regime III: With $\Delta F(z) \sim o(z^2)$, we can consider the diffusion dynamics of the nanoparticle as the random walk with double reflecting boundaries at z=-r_{in} and z=r_{in}[12]. Thus, the MSD takes the form:

$$\left\langle \Delta z^2(t) \right\rangle = \frac{r_{\text{in}}^2}{6}\left(1-\frac{96}{\pi^4}\sum_{p=0}^{\infty}\frac{1}{(2p+1)^4}\exp\left[-\frac{\pi^2}{4}(2p+1)^2\frac{t}{\tau_0}\right]\right) \tag{S62}$$

where $\tau_0 = m/\gamma$. In the long-time limit, it reduces to $\left\langle \Delta z^2 \right\rangle = r_{\text{in}}^2/6$. Using the series $\sum_{p=0}^{\infty}\frac{1}{(2p+1)^4}=\frac{\pi^4}{96}$, $\sum_{p=0}^{\infty}\frac{1}{(2p+1)^2}=\frac{\pi^2}{8}$, the Eq. S62 in the short-time limit ($t \ll \tau_0$) reads

$$\left\langle \Delta z^2(t) \right\rangle \approx \frac{r_{\text{in}}^2}{6}\left[1-\exp(-\frac{t}{\tau_0})\right] \tag{S63}$$

IV. Topology-mediated oscillation modes

In this section, we evaluate the effect of the inertia term on the oscillation modes. The nanoparticle's motion results from the balance of the frictional force, stochastic 'noise' and the potential force, as indicated by the nonlinear Langevin equation (NLE) Eq. S28. Taking into consideration of the inertia term d^2z/dt^2 , the influence of 'noise' can be eliminated by ensemble average method[11], leading to

$$\frac{d^2\left\langle z^2(t)\right\rangle}{dt^2}+2\frac{\gamma}{m}\frac{d\left\langle z^2(t)\right\rangle}{dt}+2\left\langle \Delta F'(z)\right\rangle = 2\frac{k_BT}{m} \tag{S64}$$

It is noteworthy that the solution of the second-order differential Eq. S64 has the form of $\sim e^{-(v+i\Omega)t}$, depending on the damped coefficient v and the characteristic frequency of oscillation Ω. As schemed in Fig. 5c in the main text, if a system is damped without oscillatory response, i.e., $|v|>0$, $|\Omega|=0$, the oscillation mode is overdamped; if a system is damped with oscillatory

response, i.e., $|v|>0$, $|\Omega|>0$, the oscillation mode changes into underdamped; if a system is undamped with oscillatory response, i.e., $|v|=0$, $|\Omega|>0$, the oscillation mode becomes harmonic. However, if the inertia effects are negligible by setting $d^2z/dt^2=0$ in Eq. S28, Eq. S64 can be transformed into

$$\frac{\gamma}{m}\frac{d\langle z^2\rangle}{dt}+\langle \Delta F'(z)\rangle=\frac{k_BT}{m} \tag{S65}$$

The solution of which takes the purely exponential form, e^{-vt}, resulting in the overdamped mode of oscillation as prevailed in Brownian dynamics.

As discussed in the above section, the diffusion dynamics of Regimes I, II and III exhibit various oscillation modes, highly correlating to the forms of the free energy landscape $\Delta F(z)$ in Eq. (S64). Specifically, in Regime I, the solution Eq. S50 satisfies $|v|$, $|\Omega|>0$, corresponding to the underdamped mode of oscillation. In Regime II, the mode relies on the competition between the dynamic viscosity γ/m and the frequency ω: for $\gamma>\sqrt{8}m\omega$, Eq. S59 has $|v|>0$, $|\Omega|=0$, giving rise to the overdamped mode of oscillation; for $\gamma<\sqrt{8}m\omega$, Eq. S60 has $|v|$, $|\Omega|>0$, showing the underdamped mode of oscillation. In Regime III, Eq. S62 has $|v|>0$, $|\Omega|=0$, resulting in the overdamped mode of oscillation.

Consequently, the topological-mediated oscillation modes of the nanoparticle in macromolecular networks can be rationalized based on the forms of the free energy landscape in different regimes. Physically, the oscillation modes depend on the response around the point of minimum free energy at $z = 0$. In Regime I where a nanoparticle is confined in an entire cell, it is always subjected to negative response with a constant force ρ, inducing the oscillatory behavior. In Regime II, affected by partial chains of the cell, the nanoparticle turns to receiving negative response with a linear restoring force ωz, which competes with the frictional force. When the

restoring force is large, this corresponds to obvious oscillatory behavior. By contrast, when the restoring force becomes small, nanoparticle dynamics is essentially diffusive. In Regime III where the nanoparticle experiences the free energy at the boundary of the cell, the response reduces to zero. Therefore, the dynamics in Regime III is dominated only by the frictional force, and the oscillation is overdamped.

However, as only a local network loop is considered in previous works, the viscosity force of the nanoparticle is far larger than the restoring force around z = 0. Thus, oscillatory behavior of the nanoparticle is suppressed and the underdamped modes of oscillation cannot be observed.

VI. Monte Carlo (MC) simulation

For a macromolecular network with polyhedral cells, the standard coordinates $\mathbf{r}_i$ and the linker connection $\mathbf{r}_{ij}$ can be found in ref. [5]. The centers of the polyhedron $\mathbf{r}_{cent}$ and one face $\mathbf{r}_{face}$ are defined as

$$\mathbf{r}_{face} = \frac{1}{Nm}\sum_{i\in p}\mathbf{r}_i \tag{S66}$$

$$\mathbf{r}_{cent} = \frac{1}{Nn}\sum_{i=1}^{n}\mathbf{r}_i \tag{S67}$$

where p is the group of m monomers on the face, and n is the total number of monomers of the polyhedron. Thus, the distance between the center of the polyhedron and one of its faces is given by

$$z = \frac{(\mathbf{r}_{face} - \mathbf{r}_{cent})\cdot(\mathbf{r}_{np} - \mathbf{r}_{cent})}{\left|\mathbf{r}_{face} - \mathbf{r}_{cent}\right|} \tag{S68}$$

In the MC simulations, we employ the model of Kremer-Grest bead-spring chains to mimic the flexible chains between cross-links[13,14]. A cross-linked chain of N=12 bonds is identified in continuous space, with each monomer having bonded and non-bonded interactions. The Hamiltonian is given by

$$H = \sum_{i=1}^{N} U^{Bond}(l_i) + \sum_{i<j} U^{NB}(r_{ij}) \tag{S69}$$

where $U_{\mathrm{Bond}}(l_{\mathrm{i}})$ is the bonded potential as a function of bond length l_{i}, $U^{NB}(\mathbf{r}_{ij})$ represents the nonbonded energy of the pair ij, and r_{ij} is the distance between each pair of monomers. Nonbonded monomers interact via a truncated-shifted WCA potential given by

$$U^{NB}(r_{ij}) = \begin{cases} U^{WCA}(r_{ij}) - U^{WCA}(r_c) & r_{ij} \le r_c \\ 0 & r_{ij} > r_c \end{cases} \tag{S70}$$

where

$$U^{WCA}(r_{ij}) = 4\varepsilon\left[(\frac{\sigma}{r_{ij}})^{12} - (\frac{\sigma}{r_{ij}})^{6} \right] \tag{S71}$$

the cutoff distance r_c is $2^{1/6}\sigma$, and σ, ε are respectively the length and energy units of the system. Interactions between bonded monomers are described by finite extensible nonlinear elastic (FENE) potential[15,16]

$$U^{Bond}(l_i) = -0.5kR_0^2 \ln[1-(l_i/R_0)^2] \tag{S72}$$

where R_0=1.5σ is the finite extensibility, and k=30ε/σ^2 is the stiffness constant.

In the MC simulations, we apply the Wang-Landau (WL) method[13,17] to accelerate the extraction of free energy in respect to z, $F(z)$. The flat histogram or Wang-Landau sampling method, where an automatically generated bias or penalty function, $f(X^d)$, is applied to the system along d dimension coordinates, X^d, so that the configurational integral reads

$$Z(X^d) = \exp(-\beta X^d)\int d\{\mathbf{r}\}\exp[-\beta H(\{\mathbf{r}\}, X^d)] \quad \text{(S73)}$$

where $\{\mathbf{r}\}$ denotes configurational space at a given state X. For every visit to a state along the coordinate, a small penalty energy, f_0, is added to $f(X^d)$ until Z is equal for all X. Thus, during simulation the free energy landscape is flattened, while the true free energy is simply the negative of the generated bias function

$$\beta F(X^d) = -\beta f(X^d) = -\ln\int d\{\mathbf{r}\}\exp[-\beta H(\{\mathbf{r}\}, X^d)] \quad \text{(S74)}$$

In our simulations, we set $z = X^d$, and the values of z are logarithmic distribution in the range from 0 to r_{in}. At least fifty independent runs are performed for each parameter set, so that the standard error is estimated within $0.3k_BT$.

VI. Supporting figures

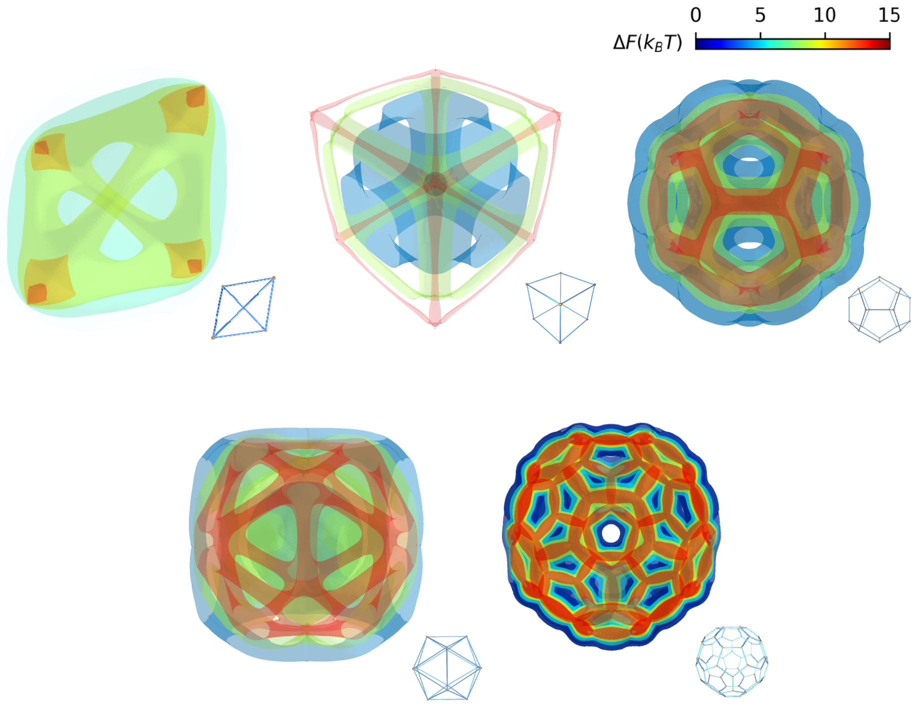

Fig. S1. Representative isosurfaces of the free energy landscape for different topologies. Isosurfaces of the free energy change for the network cells with g = 4, 6, 12, 20, 32 and d/a_x = 1.4, where the topologies of networks are schematically marked at the right bottom. The color bar on the top right corner encodes the value of ΔF.

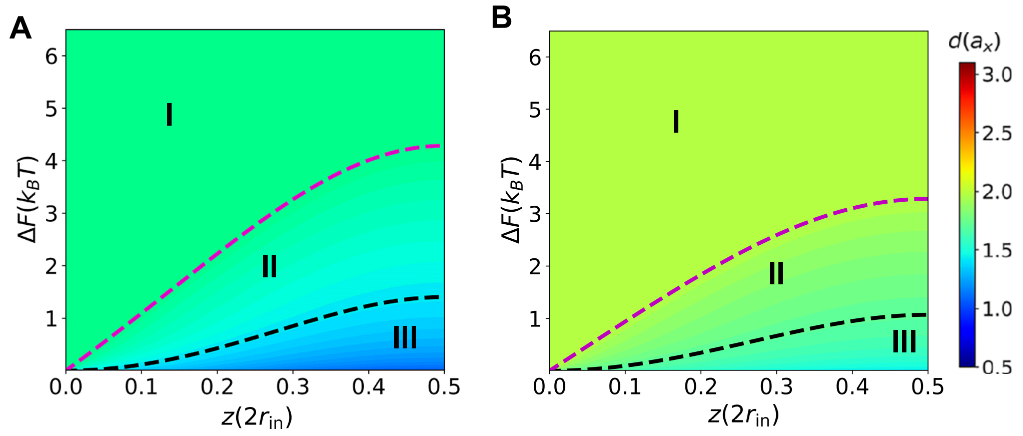

Fig. S2. Free energy change $\Delta F \sim z$ **for different topologies.** The heat map of $\Delta F \sim z$ for various d in networks at (**a**) $g = 6$, and (**b**) $g = 12$. The boundaries between regimes I and II and between regimes II and III are represented by purple and black dashed lines, respectively. The color bar indicates the values of the diameter d. The hidden regime IV in each plot gives $\Delta F(z) = 0$.

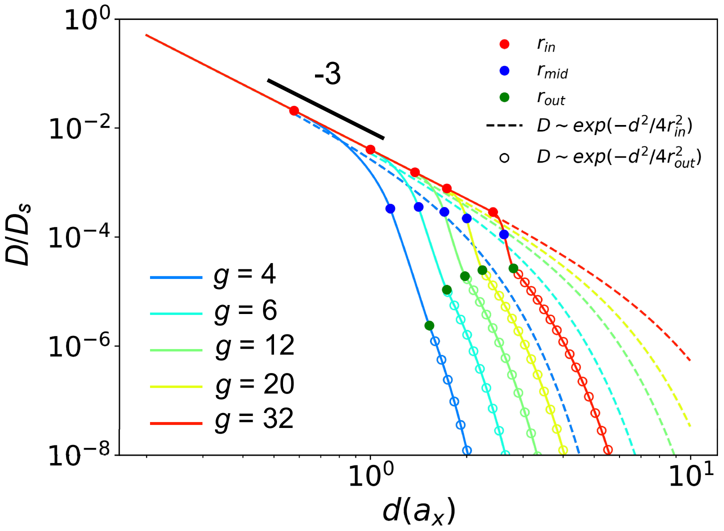

Fig. S3. Dynamics of the nanoparticle diffusion for different cell topologies. Plots of the diffusion coefficient D/D_s against d/a_x for various cell topologies, where D_s corresponds to the diffusion coefficient of the solvent. The geometrical parameters determining the topological anisotropy are presented on each plot. The exponential dependence on the square of the ratio between d and $2r_{\text{in}}$ (dashed line) or $2r_{\text{out}}$ (circular scatter) are shown.

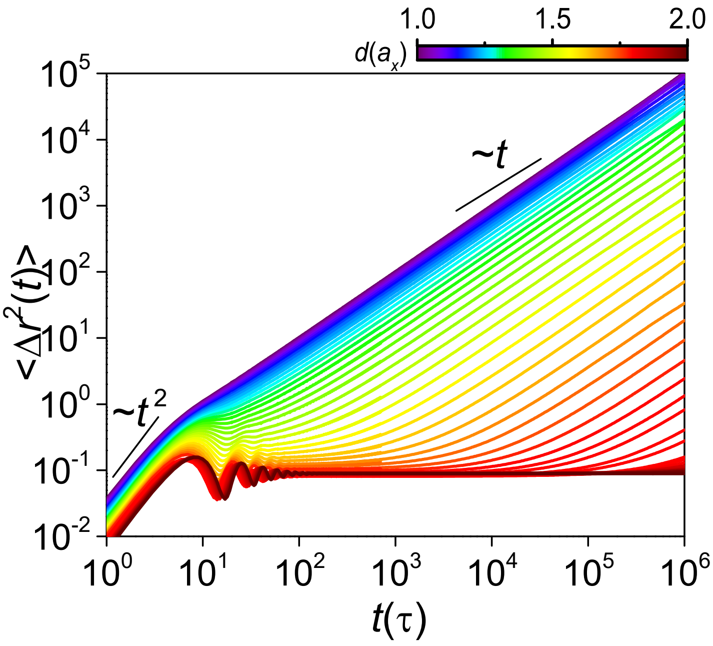

Fig. S4. The mean square displacement for several representative values of d/a_x at g=6. Numerical results of mean square displacement $<\Delta r^2(t)>$ as a function of time on log-log scales of the trajectories. The solid lines denote the numerical results, which are averaged from 10 independent runs in the total simulation time $t_c = 10^7\tau$. The color bar on the top right corner encodes the value of d.

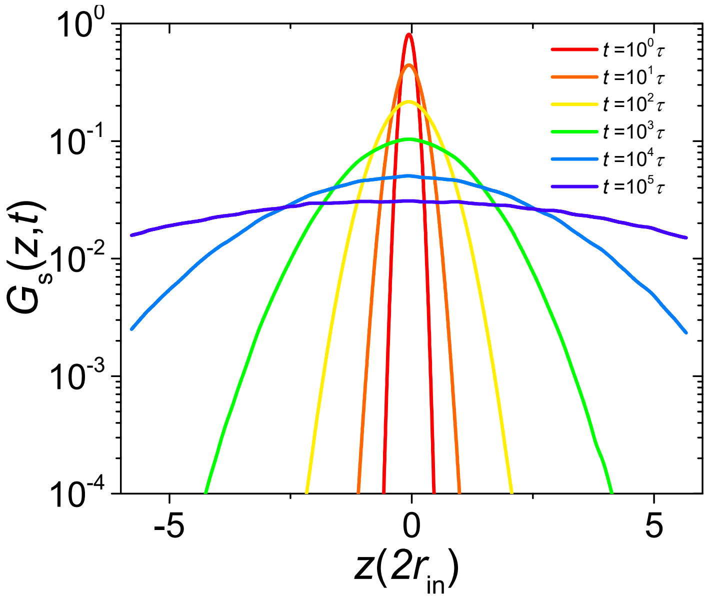

Fig. S5. The spatial displacement probability distributions $G_s(z, t)$ of Brownian dynamics.. The diameter of nanoparticle $d/a_x = 1.00$, in the macromolecular network at $g = 6$. Time t is color-coded at the top right corner.

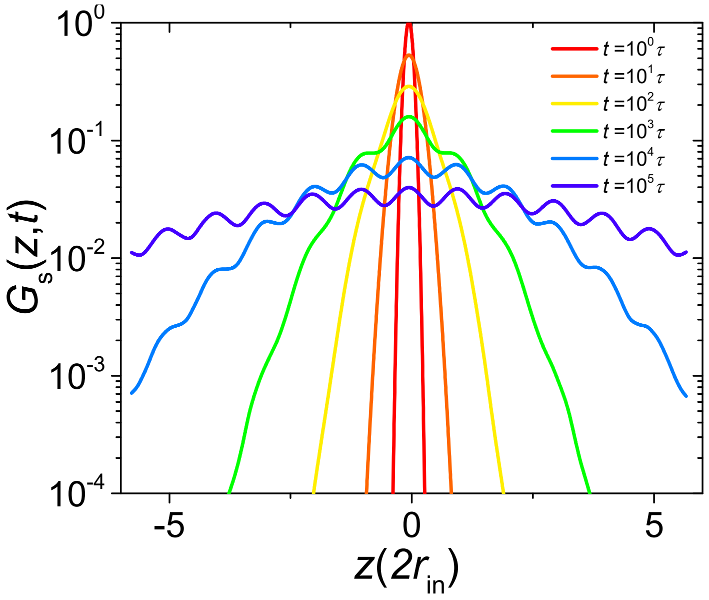

Fig. S6. The spatial displacement probability distributions $G_s(z, t)$ of hopping dynamics.. The diameter of nanoparticle $d/a_x = 1.65$, in the macromolecular network at $g = 6$. Time t is color-coded at the top right corner.

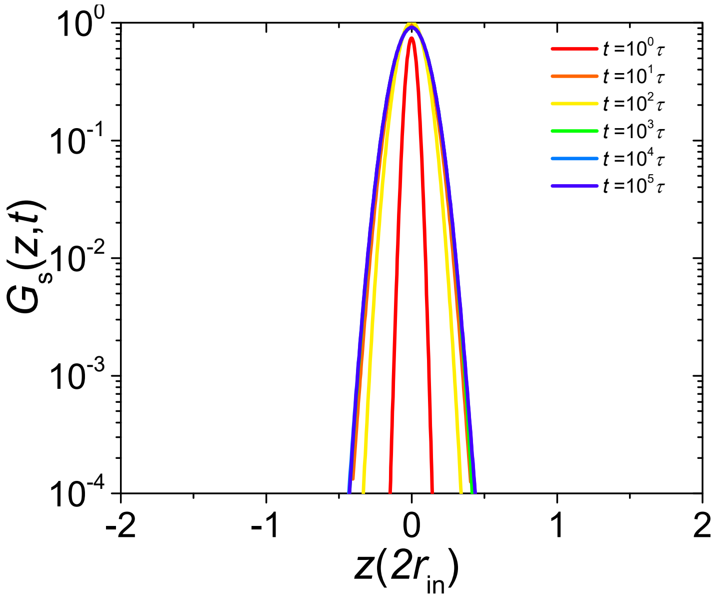

Fig. S7. The spatial displacement probability distributions $G_s(z, t)$ of trapped dynamics.. The diameter of nanoparticle $d/a_x = 1.90$, in the macromolecular network at $g = 6$. Time t is color-coded at the top right corner.

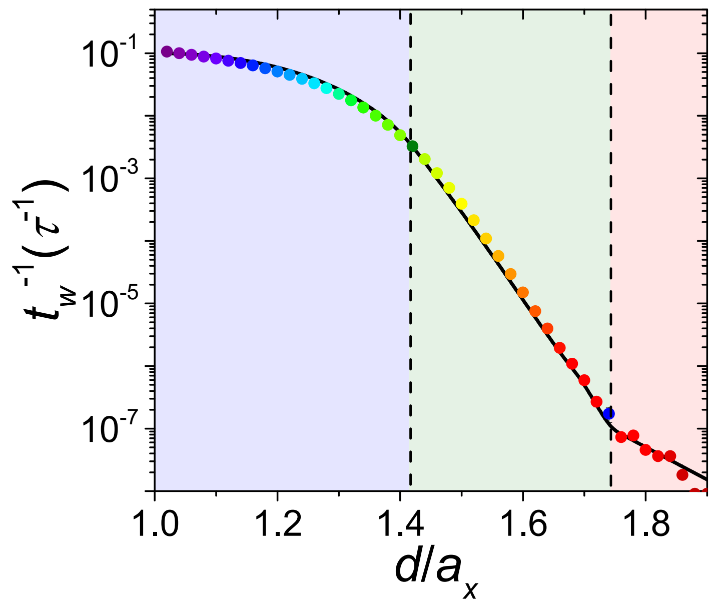

Fig. S8. The reciprocal characteristic waiting time t_w^{-1} as a function of d/a_x for the cubic cell. The characteristic waiting time t_w represents the time for nanoparticle escaping the network cell. The solid line denotes the theoretical results predicted by Eq. S40, and the dashed scatters mark the results obtained from numerical simulations by Langevin dynamics. Regimes I, II and III are presented as red, green and blue shaded sections, respectively. The boundaries between Regimes I and II and between Regimes II and III are represented by dashed lines at $d = 2r_{\text{mid}}$ (blue circle) and $d = 2r_{\text{out}}$ (green circle).

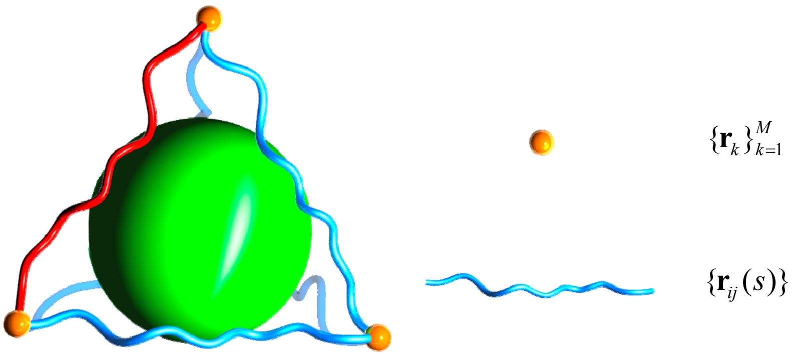

Fig. S9. Schematic of macromolecular strands in contact with a spherical nanoparticle in a network cell. Typical states in top view along the mean energy path (MEP) are illustrated in the network with g = 4 and d = 0.8a_x. The set of curve path of strands $\{\mathbf{r}_{ij}(s)\}$ are represented by cyan and red strings; and the cross-linked points $\{\mathbf{r}_i\}_{i=1}^{M}$ are represents by blue spheres, where s is the contour parameter and M is the total number of cross-links in the particle-network system. The green sphere displays the spherical particle.

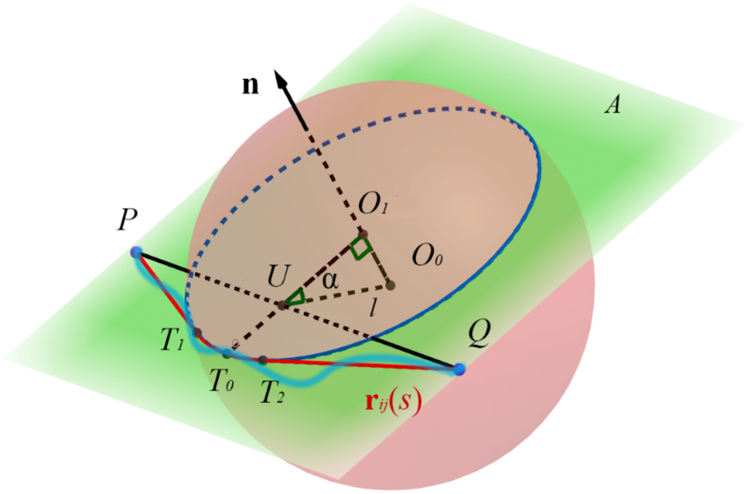

Fig. S10. Geometrical relationship of a macromolecular strand in contact with a spherical nanoparticle. The path of a strand (marked in red) $\mathbf{r}_{ij}(s)$ crosses over the surface of the spherical nanoparticle with its origin O_0. O_1 is the intersected circle(blue) of the sphere and the plane A with a normal vector $\mathbf{n}$. P, Q are the cross-linked points of the strand, and T_0, T_1 and T_2 are tangent points of P, Q and O_1 in the intersected circle. l represents the distance from O_1 to the strand PQ, and α is the dihedral angle.

VII. Supplementary references

1. Brochard-Wyart, F., & de Gennes, P. G. Dynamical scaling for polymers in theta solvents, *Macromolecules* **10**, 1157–1161 (1977).
2. Deam, R. & Edwards, S. F. The theory of rubber elasticity, *Phys. Trans. R. Soc.* **280**, 317–353 (1976).
3. Schmid, F. Self-consistent field approach for cross-linked copolymer materials, *Phys. Rev. Lett.* **111**, 028303 (2013).
4. Spakowitz, A. J. & Wang, Z. G. Semiflexible polymer confined to a spherical surface. *Phys. Rev. Lett.* **91**, 166102 (2003).
5. Cundy H. M. & Rollett A. P. *Mathematical Models* (Clarendon Press, 1952).
6. Lemons, D. S. *An Introduction to Stochastic Processes in Physics*, (The Johns Hopkins University Press, 2002).
7. Montroll, E. W. & Weiss, G. H. Random walks on lattices. II. *J. Math. Phys.* **6**, 167–181 (1965).
8. Cohen, A. E. Control of nanoparticles with arbitrary two-dimensional force fields. *Phys. Rev. Lett.* **94**, 118102 (2005).
9. Singh, R. K., Metzler, R., & Sandev, T. Resetting dynamics in a confining potential. *J. Phys. A-Math. Theor.* **53**, 505003 (2020).
10. Uhlenbeck, G. E., & Ornstein, L. S. On the theory of the Brownian motion. *Phys. Rev.* **36**, 823 (1930).
11. Contreras-Vergara, O., Lucero-Azuara, N., Sánchez-Salas, N., & Jiménez-Aquino, J. I. Harmonic oscillator Brownian motion: Langevin approach revisited. *Rev. Mex. Fis. E* **18**, 97–106 (2021).

12. Kusumi, A., Sako, Y., & Yamamoto, M. Confined lateral diffusion of membrane receptors as studied by single particle tracking (nanovid microscopy). Effects of calcium-induced differentiation in cultured epithelial cells. *Biophys. J.* **65**, 2021–2040 (1993).

13. Seaton, D. T., Schnabel, S., Landau, D. P., & Bachmann, M. From flexible to stiff: systematic analysis of structural phases for single semiflexible polymers. *Phys. Rev. Lett.* **110**, 028103 (2013).

14. Kremer, K., & Grest, G. S. Dynamics of entangled linear polymer melts: A molecular - dynamics simulation. *J. Chem. Phys.* **92**, 5057‐5086 (1990).

15. Doye J., Sear R. P., & Frenkel D. The effect of chain stiffness on the phase behaviour of isolated homopolymers. *J. Chem. Phys.* **108**, 2134–2142 (1998).

16. Ivanov V. A., Paul W., & Binder K. Finite chain length effects on the coil–globule transition of stiff-chain macromolecules: A Monte Carlo simulation. *J. Chem. Phys.* **109**, 5659–5669 (1998).

17. Wang F. & Landau D. P. Efficient, multiple-range random walk algorithm to calculate the density of states. *Phys. Rev. Lett.* **86**, 2050 (2001).
